# Early Growth Response Gene 2-Expressing CD4^+^LAG3^+^ Regulatory T Cells: The Therapeutic Potential for Treating Autoimmune Diseases

**DOI:** 10.3389/fimmu.2018.00340

**Published:** 2018-02-26

**Authors:** Tomohisa Okamura, Kazuhiko Yamamoto, Keishi Fujio

**Affiliations:** ^1^Department of Allergy and Rheumatology, Graduate School of Medicine, The University of Tokyo, Tokyo, Japan; ^2^Max Planck-The University of Tokyo Center for Integrative Inflammology, The University of Tokyo, Tokyo, Japan; ^3^Laboratory for Autoimmune Diseases, Center for Integrative Medical Sciences, RIKEN, Yokohama, Japan

**Keywords:** Egr2, Egr3, lymphocyte activation gene 3, Foxp3, regulatory T cell, gene therapy, cell therapy

## Abstract

Regulatory T cells (Tregs) are necessary for the maintenance of immune tolerance. Tregs are divided into two major populations: one is thymus derived and the other develops in the periphery. Among these Tregs, CD4^+^CD25^+^ Tregs, which mainly originate in the thymus, have been extensively studied. Transcription factor *Foxp3* is well known as a master regulatory gene for the development and function of CD4^+^CD25^+^ Tregs. On the other hand, peripheral Tregs consist of distinct cell subsets including Foxp3-dependent extrathymically developed Tregs and interleukin (IL)-10-producing type I regulatory T (Tr1) cells. Lymphocyte activation gene 3 (LAG3) and CD49b are reliable cell surface markers for Tr1 cells. CD4^+^CD25^−^LAG3^+^ Tregs (LAG3^+^ Tregs) develop in the periphery and produce a large amount of IL-10. LAG3^+^ Tregs characteristically express the early growth response gene 2 (*Egr2*), a zinc-finger transcription factor, and exhibit its suppressive activity in a Foxp3-independent manner. Although *Egr2* was known to be essential for hindbrain development and myelination of the peripheral nervous system, recent studies revealed that *Egr2* plays vital roles in the induction of T cell anergy and also the suppressive activities of LAG3^+^ Tregs. Intriguingly, forced expression of *Egr2* converts naive CD4^+^ T cells into IL-10-producing Tregs that highly express LAG3. Among the four *Egr* gene family members, *Egr3* is thought to compensate for the function of *Egr2*. Recently, we reported that LAG3^+^ Tregs suppress humoral immune responses *via* transforming growth factor β3 production in an Egr2- and Egr3-dependent manner. In this review, we focus on the role of *Egr2* in Tregs and also discuss its therapeutic potential for the treatment of autoimmune diseases.

## Introduction

Autoimmunity can cause a broad range of human diseases. This pathology is observed in at least 5% of the general population (1). To maintain self-tolerance, immune systems evolved mechanisms that discriminate between self and non-self and respond to infection by pathogens such as viruses, bacteria, and parasites, while maintaining unresponsiveness to self-antigens. However, the discrimination between self and non-self is disrupted in some individuals and the immune system is misdirected to attack self-antigens. These conditions can affect one or many different types of organs in the body.

Immune tolerance is maintained by many regulatory cell populations, including CD4^+^ regulatory T cells (Tregs) (2), CD8^+^ Tregs (3), regulatory B cells (4), dendritic regulatory cells (5), and regulatory macrophages (6). Among these regulatory cells, the CD4^+^ Treg subset, which has a pivotal role in the control of self-tolerance and inflammatory responses, is the most extensively studied in the context of autoimmunity. CD4^+^ Tregs are divided into two main subsets: one includes thymus-derived, naturally occurring Tregs (nTregs), and the other develops in the periphery. nTregs co-express interleukin (IL)-2 receptor α (CD25) and the transcription factor Forkhead box P3 (Foxp3) protein (7). *Foxp3* is a crucial gene for the development and regulatory function of CD4^+^CD25^+^ Tregs (CD25^+^ Tregs). The identification of both surface markers and a master regulatory transcription factor has significantly contributed to our understanding of molecular suppressive mechanisms of Tregs. These thymus-derived Tregs (tTregs) can expand in the periphery and exert their antigen-specific suppressive activities to maintain immune tolerance (8, 9).

The majority of the CD4^+^ Treg subset develops in the periphery, and they likely exert their suppressive activities *via* a Foxp3-independent manner. An experiment of adoptive transfer of CD4^+^Foxp3^−^ cells into non-lymphopenic hosts suggested that peripheral conversion could account for approximately 4–7% of Foxp3^+^ Tregs (10). Other group reported that Foxp3^+^ Tregs developed in the periphery comprise ~15% of the peripheral Foxp3^+^ Tregs (11). These peripherally derived Tregs (pTregs) are thought to play a distinct role in controlling adaptive immunity to restrain allergic inflammation at mucosal surfaces (12).

The lack of specific markers that can reliably distinguish Foxp3-independent Tregs from other T cell populations makes it difficult to assess their suppressive mechanisms. In 2009, we identified a Foxp3-independent IL-10-producing Treg subset, i.e., CD4^+^CD25^−^Foxp3^−^ T cells. These cells characteristically express both the lymphocyte activation gene 3 (Lag3) and the transcription factor early growth response gene 2 (Egr2) (13).

In a broad range of autoimmune diseases, these Treg subsets are impaired and decreased in frequency. Therefore, many approaches have been examined to expand functional Treg subsets both *in vitro* and *in vivo*. Gene modification of CD4^+^ T cells could be used to induce Treg subsets for therapeutic intervention in autoimmune diseases. During the past decade, a numbers of murine and human studies have investigated the therapeutic potential of *Foxp3* gene transduction in CD4^+^ T cells. The present review focuses on the molecular features of *Egr2* in Tregs and discusses the prospects and obstacles to the clinical development of gene modified Treg cell therapy.

## Nomenclature of CD4^+^ Tregs

The discovery of the role of Foxp3 is considered the most important finding in Treg biology. Deficiency of the *Foxp3* gene abrogates self-tolerance and causes autoimmune disease (14). Scurfy mice, which have a frame shift mutation in the *Foxp3* gene, fail to generate thymus-derived, nTregs and display extensive severe inflammatory infiltration in multiple organs such as the lung, skin, and liver (15). Immunodysregulation, polyendocrinopathy, enteropathy, and X-linked (IPEX) syndrome, which is caused by mutations in the *FOXP3* gene, is characterized by neonatal autoimmune type 1 diabetes, polyendocrinopathy, autoimmune hemolytic anemia, autoimmune enteropathy, and skin rash (16). A common feature of scurfy mice and IPEX syndrome is a severe deficiency of CD25^+^ Tregs. Thus, Foxp3 is considered the “master regulator” of CD25^+^ Tregs. With regard to Foxp3-dependency, Foxp3-dependent Tregs can be divided into three populations (17): first are tTregs, also known as thymus-derived nTreg. Second, Foxp3^+^ Tregs that differentiate in the periphery from Foxp3^−^ conventional CD4^+^ T cells are termed “peripherally derived Tregs.” The nomenclature for these two Foxp3^+^ Tregs populations is clearly based on the anatomical locations of their differentiation. Although it has been widely assumed that freshly isolated Foxp3^+^ Tregs mainly consist of tTregs, the ratio of tTregs to pTregs has not been completely clarified (18). Third, Foxp3^+^ Tregs generated *ex vivo* are defined as “*in vitro*-induced Treg (iTreg).” Foxp3 can be upregulated upon T cell receptor (TCR) stimulation of peripheral naive CD4^+^ T cells in the presence of transforming growth factor (TGF)-β1 (19). Although the term “iTreg” is also widely used to define “extrathymically generated Tregs in the periphery” regardless of Foxp3 dependency, we use the term “iTreg” as “Foxp3^+^ Treg generated *ex vivo*” in the present review.

On the other hand, Foxp3-independent Treg subsets are thought to develop in the periphery. These Treg subsets consist of heterogeneous subsets, including IL-10-producing CD4^+^ type I Tregs (Tr1 cells) (20) and iTr35 (21). Tr1 cells are defined by a unique cytokine production profile consisting of high levels of IL-10 and their ability to suppress immune responses through IL-10 production in a Foxp3-independent manner (20). Tr1 cells also produce variable amounts of IL-5, GM-CSF, and IFN-γ and minimal amounts of IL-2, IL-4, and IL-17 (22, 23). Although cytokine profiles of Tr1 cells, such as TGF-β1, IFN-γ, and IL-5, are dependent on experimental conditions, IL-10 production is thought to be the true hallmark of Tr1 cells (24). Production of IL-4 is consistently undetectable, which is distinct from Th2 cells. In this review, in accordance with conventional nomenclature, we mainly use the general term “Tr1 cells” for both “peripherally derived Tr1 cells (pTr1 cells)” and “*in vitro*-induced Tr1 cells (iTr1 cells).”

The Foxp3-dependent Tregs, including tTreg, pTreg, and iTreg, and Foxp3-independent extrathymically developed Tregs such as Tr1 cells are thought to be fundamental for maintaining adequate immune tolerance.

## IL-10-Producing CD4^+^CD25^−^LAG3^+^ Tregs

Interleukin-10 is an anti-inflammatory cytokine that is produced by a wide range of cell types, including not only CD4^+^ cells but also CD8^+^ T cells, B cells, dendritic cells, macrophages, mast cells, natural killer cells, eosinophils, and neutrophils during the course of immune responses (25). As for CD4^+^ Th cells, IL-10 was first described as a product of Th2 cells that suppressed cytokine secretion from Th1 cells (26). Th1 cells also produce IL-10 *via* ERK1 and ERK2 MAP kinase phosphorylation and IL-12-induced transducer and activator of transcription (STAT) 4 activation (27). IL-10 production from Th17 cells exerts tissue-protective and immunosuppressive effects (28). During infections, IL-10 production from these Th cell subsets might be an essential mechanism underlying the self-limitation that dampens excessive immune responses and prevents tissue damage (29).

Production of IL-10 is closely related to the function of Treg subsets. IL-10-producing Tregs can be developed *in vivo* in both Foxp3-dependent and Foxp3-independent manners (30). To date, two major subsets of IL-10-producing Tregs have been identified; one subset includes Foxp3^+^ Tregs and the other is represented by Foxp3-independent Tr1 cells generated extrathymically. However, Foxp3^+^ Tregs do not produce IL-10 following stimulation after *ex vivo* isolation, unless isolated from the gut. Foxp3^+^ Tregs inhibit naive T cell proliferation *in vitro* in an IL-10-independent manner. In contrast, Foxp3^+^ Tregs exert their suppressive activity *in vivo* in an IL-10-dependent manner, suggesting that Foxp3^+^ Tregs need signals *in vivo* to induce IL-10 (25). On the other hand, the best characterized Foxp3-independent, IL-10-producing Tregs are Tr1 cells. Although other Th subsets also produce IL-10 (see above), Tr1 cells produce greater amounts of IL-10 shortly after activation compared to other Th subsets (31, 32). Andolfi et al. demonstrated that forced expression of IL-10 by human CD4^+^ T cells confers the phenotype and function of Tr1 cells (2).

**Figure 1:**
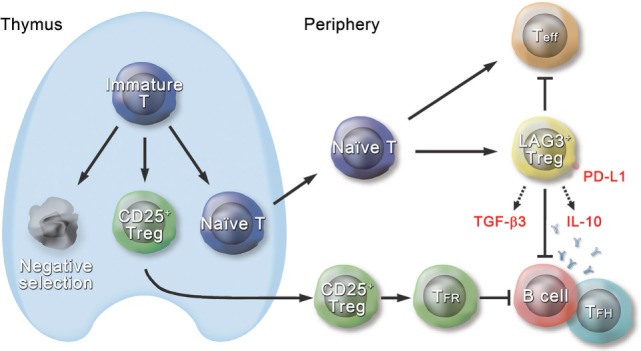
Schematic diagram of the role of CD4^+^CD25^−^LAG3^+^ regulatory T cells (Tregs) in the immune system. Approximately 2% of the CD4^+^CD25^−^ T cell population in the spleen consists of CD4^+^CD25^−^LAG3^+^ T cells (LAG3^+^ Tregs), which are extrathymic in origin. LAG3^+^ Tregs characteristically express transcription factor early growth response gene 2 (Egr2) and produce large amounts of interleukin (IL)-10 and transforming growth factor (TGF)-β3 in response to antigenic stimulation. Ectopic expression of Egr2 by naive CD4^+^ T cells confers the phenotype of LAG3^+^ Tregs, including expression of Prdm1 and IL-10. LAG3^+^ Tregs suppress effector T cells (Teff) in an IL-10-dependent and Foxp3-independent manner. Like thymus-derived CD4^+^CD25^+^Foxp3^+^CXCR5^+^ Tregs, called follicular regulatory T (T_FR_) cells, LAG3^+^ Tregs control germinal center responses. Although T_FR_ cells express CXCR5 and exist in the germinal center (GC), LAG3^+^ Tregs do not express CXCR5 protein and are located at the T-B border in the steady state. Suppression of GC responses by LAG3^+^ Tregs is mediated by TGF-β3, and Egr2 is essential for effective secretion of TGF-β3 from LAG3^+^ Tregs. TGF-β3-mediated B cell suppression requires programmed cell death 1 (PD-1) expression on B cells, and on the other hand, LAG3^+^ Tregs highly express PD-1 ligand (PD-L1). Thus, LAG3^+^ Tregs are equipped with various mechanisms to maintain the immune tolerance.

Type I Tregs have been a focus of active investigation. Nonetheless, until recently, it has been difficult to assess their *in vivo* physiological function because of the lack of specific cell surface markers and master regulatory genes. However, in 2009, we identified IL-10-producing CD4^+^CD25^−^ Tregs that characteristically express cell surface marker LAG3 and transcription factor Egr2 (13). Approximately 2% of the CD4^+^CD25^−^ T cell population in the spleen consisted of CD4^+^CD25^−^LAG3^+^ T cells (LAG3^+^ Tregs) (Figure 1). Unlike tTregs, high-affinity interactions with self-peptide/major histocompatibility complex ligands expressed in the thymus are not necessary for the development of LAG3^+^ Tregs. Those results indicate that LAG3^+^ Tregs are extrathymic in origin. LAG3^+^ Tregs, which do not express Foxp3 protein, secrete higher levels of IL-10 than do CD25^+^ Tregs. In addition, LAG3^+^ Tregs are hypoproliferative in response to TCR stimulation. Moreover, they suppress the *in vivo* development of colitis induced in RAG-1^−/−^ recipients by the transfer of naive CD4^+^ T cells in an IL-10-dependent manner. LAG3^+^ Tregs from Scurfy mice still express *IL10* mRNA and retain regulatory activity *in vitro*. These findings indicate that LAG3^+^ Tregs are equivalent to pTr1 cells that exist in a steady state.

Groux et al. first reported a unique cytokine production profile of Tr1 cells induced *in vitro*, i.e., IL-10^+^ IL-4^−^ IL-5^+^ TGF-β1^+^ IL-2^low/−^ IFN-γ^low/−^ (23). Th3 regulatory cell induced *in vivo* is a potent source of TGF-β1, IL-10, and IL-4 protein (33). However, LAG3^+^ Tregs do not produce TGF-β1, IL-4, and IL-5 protein (13). Thus, although LAG3^+^ Tregs fulfill the definition of Tr1 cells (24, 34), further studies are required to elucidate the inconsistency between these IL-10-producing Tregs induced *in vitro* or *in vivo* and LAG3^+^ Tregs, which exist in a steady state, in more detail. Recently, Gagliani et al. reported that concomitant expression of LAG3 and CD49b is specific for Tr1 cells in humans and mice (35). Subsequent findings in antigen-specific immunotherapy based on the administration of cognate peptides in escalating dose immunotherapy (EDI) indicated that the levels of LAG3 and CD49b correlated positively with IL-10 expression in CD4^+^ T cells, and LAG3 was highly upregulated and was maintained during EDI treatment (36). Although *Lag3* mRNA is expressed on CD4^+^CD25^+^Foxp3^+^ Tregs (37), LAG3 protein was hardly detected on the cell surface of CD4^+^CD25^+^ Tregs (38). These observations indicate that LAG3 and CD49b are phenotypic markers of IL-10-producing, Foxp3-independent, extrathymically induced CD4^+^ Tregs that play a major role in regulating the activity of the immune system (39).

Interleukin-10 receptor is expressed on a variety of immune cells (40). IL-10-deficient mice develop an inflammatory bowel disease characterized by dysregulated production of pro-inflammatory cytokines (41) and show a prolonged fever in response to lipopolysaccharide (42). Deficiency of IL-10 exacerbates autoimmune pathology in mouse models of systemic lupus erythematosus (SLE) (43), rheumatoid arthritis (RA) (44), and experimental autoimmune encephalomyelitis (EAE) (45), indicating the critical role of IL-10 in the regulation of immune homeostasis. Thus further studies on LAG3^+^ Tregs could provide insights into the development of new therapeutic targets for autoimmune diseases.

## Role of Egr2 in IL-10-Producing Tregs

Differential gene expression profiles of LAG3^+^ Tregs, CD25^+^ Tregs, CD4^+^CD25^−^LAG3^−^ T cells and naive CD4^+^CD25^−^CD45RB^high^ T cells have been constructed by gene array analysis. The data revealed that transcription factor Egr2 were preferentially expressed in LAG3^+^ Tregs (13). The Egr family consists of four Cys2His2-type zinc-finger transcription factors, Egr-1, -2, -3, and -4. Egr2 plays an essential role in hindbrain development and myelination of the peripheral nervous system. Egr2 deficiency results in perinatal or neonatal death due to respiratory and/or feeding deficits (46). During the last decade, the role of Egr2 in T cells has been extensively elucidated. Egr2 is necessary for the TCR-induced negative regulatory program because it controls the expression of the E3 ligase Cbl-b, which is critical for the regulation of T cell tolerance and anergy (47, 48). Zhu et al. revealed that Egr2 binds directly to the promoter of the cell cycle inhibitor p21^cip1^ in T cells (49).

Our previous study revealed the role of Egr2 in Tregs (13). In the steady state, LAG3^+^ Tregs, but not Foxp3^+^ Tregs, specifically expressed not only Egr2 but also IL-10 and Blimp-1, which is a critical regulator for IL-10 production from Th subsets (50–52). To determine whether forced expression of Egr2 in naive CD4^+^ T cells could convert them to a LAG3^+^ Treg phenotype, we constructed retroviral vectors that co-expressed green fluorescent protein (GFP) and Egr2. As expected, the Egr2-transduced GFP^+^CD4^+^ cells showed significant upregulation of LAG3 and Blimp-1. In addition, these Egr2-transduced GFP^+^CD4^+^ cells produced high levels of IL-10 and lower amounts of IL-2, IL-4, and IL-5 proteins, resembling the cytokine profile seen in Tr1 cells (20). We confirmed that the ectopic expression of Egr2 conferred a suppressive function on activated CD4^+^ T cells (13). These observations clearly demonstrated the critical importance of Egr2 for IL-10 expression in LAG3^+^ Tregs.

Early growth response gene 2 is expressed at high levels in LAG3^+^ Tregs. In contrast, it is barely expressed in other T cell subsets in the steady state (13, 53). Egr2 is induced in T cells after TCR stimulation (54). *In vitro*, Egr2-deficient T cells proliferate normally in response to TCR stimulation. However, Egr2-deficient T cells are hyperresponsive to exogenous IL-2, indicating that Egr2 does not affect TCR signaling, but controls the subsequent expansion of activated T cells (49). Such Egr2-deficient T cells produce higher levels of effector cytokines such as IFN-γ and IL-17. Recently, Du et al. demonstrated that Egr2 plays a pivotal role in T cells’ response to influenza virus infection by directly binding to *Tbx21* gene and promoting the expression of T-bet (55). In contrast, another group showed that Egr2 is not required for T cell responses to *Toxoplasma gondii* or lymphocytic choriomeningitis virus (56). On the other hand, Miao et al. reported that Egr2 directly interacts with Batf, a transcription factor that regulates both IL-17 production and Th17 differentiation and blocks its binding to the *IL-17* promoter (57). As for follicular helper T (T_FH_) cells, during viral infection, Egr2 and Egr3 directly regulate the expression of Bcl6 and differentiation of T_FH_ cells (58). Interestingly, despite increased effector function of Egr2-deficient T cells as described above, IL-2 production and proliferation of T cells from Egr2- and Egr3-deficient T cells are impaired (59). More recently, Miao et al. revealed that Egr2 and Egr3, induced in activated T cells, suppress T cell activation and differentiation but promote clonal expansion of virus-specific T cells (53). These observations indicate that Egr2 and Egr3 have distinct function in homeostatic condition and infection.

## IL-27 Induces Egr2 Expression

Interleukin-27, a member of the IL-12 cytokine family, plays a critical role in the development of Tr1 cells and the resolution of inflammation (60). IL-27 is a heterodimeric cytokine composed of subunit proteins IL-27p28 and EBV-induced protein 3 (61). Deficiency of IL-27 signaling results in significant reduction of IL-10-producing T cells during autoimmune disease and infection (60). IL-27 receptor signaling induces expression of cMaf and aryl hydrocarbon receptor, both of which transactivate the *IL10* and *IL21* promoters (62). Recently, Karwacz et al. reported that interferon regulatory factor 1 and BATF were rapidly induced after treatment with IL-27 and were necessary for the differentiation and function of Tr1 cells *in vitro* and *in vivo* (63). Both transcription factors were critical for preparing the chromatin landscape during Tr1 differentiation. We previously demonstrated that Egr2 mediates IL-27-induced IL-10 production in CD4^+^ T cells (50). Egr2 was induced by IL-27 in a STAT3-dependent manner and directly bound to the promoter region of *Prdm1*, encoding Blimp-1, and enhanced its activity. When cells were deficient for Egr2, IL-27 failed to induce Blimp-1 and IL-10 in CD4^+^ T cells. These observations support the essential role of Egr2 expression in the induction of IL-10 in CD4^+^ T cells.

## Egr2 and Autoimmune Diseases

In humans, mutations in *Egr2* cause Charcot–Marie–Tooth disease type 1, Dejerine–Sottas syndrome, and congenital hypomyelination neuropathy (64). Recent genome-wide association studies (GWAS) have identified new genetic links between *Egr2* and human autoimmune diseases. Two independent GWAS investigations revealed strong association signals for Crohn’s disease (the most common form of chronic inflammatory bowel disease) on chromosome 10q21, within which *Egr2* is located (65, 66). In line with these observations, we previously demonstrated that adoptive transfer of Egr2-expressing LAG3^+^ Tregs effectively ameliorated intestinal inflammation in a murine T cell transfer model of colitis (13).

A candidate gene analysis revealed that polymorphisms in the *Egr2* gene influenced the susceptibility to SLE (67). SLE is an autoimmune disease characterized by a wide range of anticellular and antinuclear autoantibodies that affect multiple organs. SLE is induced by combinations of environmental and genetic factors (68). Initially, the survival of patients with SLE showed improvements. However, in the last two decades, no substantial improvements in patient survival have been observed (69, 70). Thus, further studies are needed to clarify the precise molecular mechanisms that are involved in the pathogenesis of SLE.

Recent studies have shown an association of the *Egr2* gene with the occurrence of lupus in mice. T cell-specific Egr2 conditional knockout (CKO) mice develop progressive lupus-like autoimmunity with no impact on the development of Foxp3-dependent CD25^+^ Tregs (49). Moreover, mice deficient for both Egr2 and Egr3 in B and T cells present lethal and early-onset systemic autoimmunity, suggesting a synergistic role for Egr2 and Egr3 in controlling B cell tolerance (59). The association between Egr2 and autoantibody-mediated systemic autoimmunity suggested a linkage between Egr2-expressing LAG3^+^ Tregs and the control of lupus activity. To clarify the role of Egr2 in T cells, we generated T-cell-specific Egr2 CKO mice (*Egr2^fl/fl^* CD4-*Cre*^+^) (71). Egr2CKO mice showed significant increases in the proportion of T_FH_ cells and germinal center B (GCB) cells and exhibited an enhanced antibody response against T cell-dependent antigens. Transfer of wild-type LAG3^+^ Tregs significantly suppressed spontaneous T_FH_ and GCB formation and inhibited aberrant antibody responses.

In lupus-prone MRL-*Fas^lpr/lpr^* (MRL/lpr) mice, adoptive transfer of LAG3^+^ Tregs from control MRL-*Fas*^+/+^ (MRL/+) mice suppresses the progression of lupus in a TGF-β3-dependent manner. Although the pro-inflammatory role of TGF-β3 was previously demonstrated by the observation that TGF-β3 and IL-6 promote pathogenic Th17 cell differentiation (72, 73), the anti-inflammatory role of TGF-β3 has attracted little attention. As for helper T-cell development, TGF-β3 is autonomously produced by Th17 cells (73). We confirmed that not only Th17 cells but also Th1 cells produced TGF-β3; however, LAG3^+^ Tregs secreted greater amounts of TGF-β3 compared with Th1 and Th17 cells. TGF-β3 suppressed the phosphorylation of STAT6, Syk, and NF-κB p65 in activated B cells, indicating that TGF-β3 inhibits important pathways for B cell functions (71). TGF-β3 also effectively suppresses human B cells (74).

Moreover, we have demonstrated that LAG3^+^ Treg-mediated B cell suppression requires programmed cell death 1 (PD-1), which provides negative co-stimulatory signals to both T cells and B cells (75, 76). The *PD1* gene has been identified as an SLE-susceptible gene (68), and its deficiency in mice promotes a lupus-like disease (77). Intriguingly, LAG3^+^ Tregs highly express PD-1 ligand, and TGF-β3 enhanced PD-1 expression by stimulated B cells. These observations indicate that TGF-β3 produced by LAG3^+^ Tregs plays a major role in the maintenance of humoral immune tolerance. As IL-10 strongly suppresses development and function of Th17 cells, IL-10 produced by LAG3^+^ Treg may counteract the pro-inflammatory aspect of TGF-β3 (78). Further studies are necessary to confirm the synergistic effects of TGF-β3 and IL-10 in the immune system.

Recently, we addressed the role of Egr2 in the induction of TGF-β3 in LAG3^+^ Tregs (79). Among the four Egr family members, Egr3 is able to partially compensate for Egr2 function (49). As expected, the absence of both Egr2 and Egr3 in T cells resulted in a significant reduction of TGF-β3 secretion from LAG3^+^ Tregs and led to earlier onset of a lupus-like syndrome compared with Egr2CKO mice. Unexpectedly, *Tgfb3* mRNA was observed in LAG3^+^ Tregs even when Egr2 and/or Egr3 were deficient. TGF-β3 undergoes complex processing steps intracellularly before its secretion from the cell surface (80, 81). After translation, TGF-β3 precursor protein is cut by furin and forms a small latent complex (SLC) that consists of mature TGF-β3 and latency associated peptide (LAP). SLCs are usually associated with latent TGF-β-binding protein (Ltbp) and secreted outside the membrane as a large latent complex. The Ltbp family consists of four members. Among them, the expression of Ltbp1–4 (82) and Ltbp3 is maintained by Egr2, and Egr3 was required for TGF-β3 secretion from LAG3^+^ Tregs. Thus, Egr2 expressed in CD4^+^ T cells has an integral role in a broad range of immunological balances.

## A Therapeutic Perspective

### Tregs for Potential Cell Therapy

Therapeutic use of Tregs to treat aberrant immune responses that cause autoimmune diseases is now an important field of investigation. For example, the Scurfy mouse phenotype is ameliorated by adoptive transfer of tTregs (83). Other approaches to adoptive Treg cell therapy using either Foxp3-dependent or Foxp3-independent Tregs have been demonstrated in various autoimmune disease mouse models, including SLE (71, 84), type 1 diabetes (85), EAE (86), inflammatory bowel disease (13, 87), and collagen-induced arthritis (CIA) (88). Adoptive Treg cell transfer is also an effective treatment for allograft rejection (89).

In RA patients, treatment with a humanized anti-IL-6R monoclonal antibody (tocilizumab) reduced circulating Th17 cells and increased pTregs (90). We have confirmed that, similar to murine LAG3^+^ Tregs, human CD4^+^CD25^−^CD45RA^−^LAG3^+^ T cells express *Egr2, IL10*, and *TGFB3* mRNAs and suppressed antibody production from B cells when co-cultured with T_FH_ cells (71). Recently, we showed that the frequency of LAG3^+^ Tregs in RA patients was lower, especially those with higher Clinical Disease Activity Index scores, compared to healthy donors. Moreover, LAG3^+^ Tregs significantly increased after 6 months of treatment with abatacept, a CTLA-4 fusion protein. *In vitro* abatacept treatment conferred LAG3 and Egr2 expression on naive CD4^+^ T cells, and abatacept-treated CD4^+^ T cells exhibited suppressive activity (91).

Collectively, these results suggest approaches for the use of Treg subsets in the treatment of human diseases. Recent clinical trials, using either Foxp3^+^ Tregs or Tr1 cells, proved the safety of Treg cell therapy and suggested possible therapeutic effects (22). However, the frequency of Tregs in the peripheral blood mononuclear cell fraction is very low. *Ex vivo*-expanded Tregs may change their suppressive phenotype posttreatment, because Xu et al. demonstrated that Tregs in the absence of TGF-β can differentiate into Th17 cells (92). Since it is also difficult to ensure the high purity of human Tregs using cell surface markers, these enriched Tregs may contain pathogenic autoreactive Th cells (93).

### Gene Transfer Therapy

Gene transfer-based induction of Tregs offers an alternative promising treatment option for autoimmune diseases. Ectopic expression of the *FOXP3* gene in naive human CD4^+^ T cells from healthy donors or IPEX syndrome patients renders the cells suppressive (94–97). In mice, therapeutic approaches using *Foxp3* gene-transduced CD4^+^ Tregs have been successful in the induction of tolerance in graft-versus-host disease (98) and some autoimmune diseases (99, 100).

Gene transfer-based therapeutic approaches combining master regulatory genes of Tregs with an antigen-specific TCR could enhance the clinical efficacy of Tregs. This approach could generate large numbers of antigen-specific Tregs and reduce undesirable global immune suppression. The potential advantages are evident, as this treatment option contrasts with traditional drugs such as steroids, other immunosuppressive agents, and biologic drugs. For example, we isolated a pair of TCR α and β genes from the paw of a mouse with CIA. We co-transduced this clonotype and the *Foxp3* gene into peripheral CD4^+^ T cells. These antigen-specific, modified Tregs effectively suppressed CIA. We also observed reductions in TNF-α, IL-17A, IL-1β expression and bone destruction even when transfer occurred after the onset of arthritis (101). In contrast, Foxp3-transduced T cells without antigen specificity did not have a therapeutic effect on CIA. Subsequently, another group demonstrated that adoptive transfer of TCRαβ and *Foxp3* gene-transduced CD4^+^ T cells suppressed T cell cytokine production and the proliferation of allergen-specific effector T cells (102).

### Therapeutic Potential of Egr2-Expressing LAG3^+^ Tregs

Our laboratory demonstrated that forced expression of Egr2, which is preferentially expressed by LAG3^+^ Tregs, in naive CD4^+^ T cells could convert them to the phenotype of LAG3^+^ Tregs (13). We investigated CD4^+^ T cells from chicken ovalbumin (OVA)-specific TCR transgenic DO11.10 mice transduced with pMIG-Egr2. These cells significantly suppressed delayed type hypersensitivity reactions against OVA compared with BALB/c CD4^+^ T cells transduced with pMIG-Egr2. Those results indicated the presence of antigen-specific suppressive activity in Egr2-transduced cells. These findings suggest that Egr2-associated Tregs, as well as Foxp3-associated Tregs, can modulate antigen-specific Treg cell therapy. We previously showed that adoptive transfer of IL-10-transduced T cells from chicken OVA-specific TCR transgenic DO11.10 mice ameliorated methylated bovine serum albumin (BSA)-induced arthritis when the arthritic joint was co-injected with OVA in addition to methylated BSA without impairing the systemic immune response to the antigen. Those experiments indicated the feasibility of an optional therapeutic approach using antigen-specific non-Treg cells transduced with regulatory effector molecules specific for Tregs such as IL-10 (103).

### Antigen-Specific Chimeric Antigen Receptor (CAR) T Regulatory Cells

There are two major concerns in ectopic expression of antigen-specific TCR. First, there is the potential for mispairing between the ectopically transduced TCR and endogenous TCR (104). Second, there are limitations to the human leukocyte antigen restriction. Elinav et al. engineered hapten-specific CAR-transduced CD4^+^CD25^+^ Tregs that effectively ameliorated colitis induced by the same hapten (105). CAR, which redirects specificity for a desired cell surface antigen, consists of antigen-recognizing variable regions (scFVs) from monoclonal antibodies, a hinge/spacer peptide, a transmembrane region, and one or more cytoplasmic signaling domains. Recently, Eyquem et al. demonstrated that a CD19-specific CAR to the TCR alpha constant locus not only resulted in uniform CAR expression in human peripheral blood T cells but also averted accelerated T-cell differentiation and exhaustion. Thus, it increased the therapeutic potency of engineered T cells (106). It appears that CAR T cell therapy provides several advantages compared to gene therapy using a combination of TCR α and β single chains.

### TGF-β as a Therapeutic Target

We previously reported that Egr2-expressing LAG3^+^ Tregs, which suppressed germinal center reactions, were enriched at the T-B border, where B cells interact with T_FH_ cells (71, 79). As described above, LAG3^+^ Tregs produce large amount of TGF-β3 and Egr2 is necessary for the effective secretion of TGF-β3 from LAG3^+^ Tregs (79). These findings showed the importance of the induction of Egr2 on T cells to maintain the humoral immune tolerance *via* TGF-β3. Although we confirmed that TGF-β1 produced similar effects as TGF-β3 on B cells, LAG3^+^ Tregs do not produce TGF-β1 (71). The isotype specific production of TGF-β might be advantage of Egr2-expressing LAG3^+^ Tregs for therapeutic approach, because TGF-β1, but not TGF-β3, promotes fibrosis (107, 108).

### Current Issues of Gene Therapy

Overall, these observations indicate the therapeutic value of genetically modified Tregs in human autoimmune diseases. A previous study suggested that integrating viral vectors do not elicit clinically evident genotoxicity in T cells, unlike hematopoietic stem cells (109). However, many issues such as cell dose, stability, plasticity, epigenetic regulation, antigen specificity, cross-reactivity of engineered TCRs, vector design, and immunological responses to the transferred gene products need to be addressed.

## Conclusion

Dysregulation of the immune system results in autoimmune diseases or allergies. Although impressive advances in the development of new drugs and optimization of therapeutic protocols are being made in these fields, many pathologies are still resistant to treatment. Many people with autoimmune diseases or organ transplantation require immune-suppressive drugs that are associated with a number of complications, including increased susceptibility to severe lethal infection. Antigen-specific cell therapy is the most physiologic means to manipulate immune responses. Adoptive immunotherapy with Tregs is entering early clinical trials to prove the safety of this novel therapeutic approach (22, 110). However, the difficulty of isolating and cloning stable Tregs involved in antigen-specific inhibition of immune responses has made it impractical. To circumvent these problems, gene therapy using master regulatory molecules and/or regulatory effector molecules of Treg subsets might be a very promising therapeutic approach. For the future development of these Treg-based therapies, further studies that delineate the exact role of each Treg subset, including tTregs, pTregs, and pTr1 cells, in each disease will be required.

## Author Contributions

TO, KY, and KF designed the outline and wrote the manuscript.

## Conflict of Interest Statement

TO received financial support or fees from Chugai and Bristol-Myers Squibb (BMS). KY received financial support or fees from AbbVie, Astellas, BMS, Daiichi-Sankyo, Mitsubishi Tanabe, Pfizer, Sanofi, Santen, Takeda, Teijin, Boehringer Ingelheim, Chugai, Eisai, Ono, Taisho Toyama, UCB, ImmunoFuture, Asahi Kasei, Janssen, and NiPPON KAYAKU. KF received financial support or fees from Astellas, BMS, Daiichi-Sankyo, Mitsubishi Tanabe, Pfizer, Santen, Takeda, Chugai, Eisai, Taisho Toyama, UCB and Janssen.
